# Contributions of loneliness to cognitive impairment and dementia in older adults are independent of other risk factors and Alzheimer’s pathology: a narrative review

**DOI:** 10.3389/fnhum.2024.1380002

**Published:** 2024-05-30

**Authors:** Barry S. Oken, Josh Kaplan, Daniel Klee, Autumn M. Gallegos

**Affiliations:** ^1^Department of Neurology, Oregon Health and Science University, Portland, OR, United States; ^2^Department of Behavioral Neuroscience, Oregon Health and Science University, Portland, OR, United States; ^3^Department of Psychiatry, University of Rochester Medical Center, Rochester, NY, United States

**Keywords:** loneliness, dementia, cognitive impairment, older adults, cognition, aging

## Abstract

Loneliness significantly contributes to cognitive impairment and dementia in older adults. Loneliness is a distressing feeling resulting from a perceived lack of social connection (i.e., a discrepancy between desired and actual social relationships), while social isolation is a related term that can be defined by number and type of social relationships. Importantly, loneliness is distinct from social isolation in that it is associated with a distressing self-perception. The primary focus of this narrative review is the impact of chronic loneliness on cognitive impairment and dementia among older adults. Loneliness has a significant association with many factors that are related to worse cognition, and therefore we include discussion on health, mental health, as well as the physiological effects of loneliness, neuropathology, and potential treatments. Loneliness has been shown to be related to development of dementia with a hazard ratio (HR) risk comparable to having a single APOE4 gene. The relationship of dementia to loneliness appears to be at least partially independent of other known dementia risk factors that are possibly associated with loneliness, such as depression, educational status, social isolation, and physical activity. Episodic memory is not consistently impacted by loneliness, which would be more typically impaired if the mild cognitive impairment (MCI) or dementia was due to Alzheimer’s disease (AD) pathology. In addition, the several longitudinal studies that included neuropathology showed no evidence for a relationship between loneliness and AD neuropathology. Loneliness may decrease resilience, or produce greater cognitive change associated with the same level of AD neuropathology. Intervention strategies to decrease loneliness in older adults have been developed but need to consider key treatment targets beyond social isolation. Loneliness needs to be assessed in all studies of cognitive decline in elders, since it significantly contributes to the variance of cognitive function. It will be useful to better define the underlying mechanism of loneliness effects on cognition to determine if it is similar to other psychological factors related to excessive stress reactivity, such as neuroticism or even depression, which are also associated with cognitive decline. It is important from a health perspective to develop better strategies to decrease loneliness in older adults.

## 1 Introduction

Loneliness is a distressing feeling resulting from a perceived lack of social connection, i.e., a discrepancy between desired and actual social relationships (Peplau and Perlman, 1982; Boss et al., 2015; Leigh-Hunt et al., 2017). The primary focus of this narrative review is the impact of chronic loneliness on cognitive impairment and dementia in older adults, and includes discussion on its impact on health, mental health, physiological effects, neuropathology, and potential treatments. Transient or situational loneliness may also occur, but it is less clearly related to cognitive decline [e.g., Akhter-Khan et al. (2021)].

Cognitive impairment is usually assessed from personal history and cognitive testing to generate a clinical diagnosis of mild cognitive impairment (MCI) or dementia, often using some additional neuroimaging or biomarker data to exclude non-neurodegenerative causes or more precisely define the diagnosis. In most epidemiological papers evaluating dementia incidence related to loneliness, there is generally just a clinical diagnosis of dementia that is attributed to Alzheimer’s disease (AD) in the absence of other obvious causes based on clinical history, neurological exam or neuroimaging, e.g., vascular dementia, fronto-temporal lobar degeneration or Lewy Body Disease. This approach is consistent with the older consensus research diagnosis of probable AD (Jack et al., 2011). However, this older definition has significant limitations and is in the process of being updated to include biomarkers (Jack et al., 2018; Alzheimer’s Association International Conference, 2023). Older adults, particularly over age 80 years, may meet the clinical definition of probable AD but either have no evidence of AD neuropathology, have multiple neuropathological findings, or have a significant amount of variance in their cognition not attributable to the usual neuropathologies (Schneider et al., 2007; Brayne et al., 2009; Boyle et al., 2013). Also, some other stressful conditions increasing likelihood of dementia possibly related to loneliness [e.g., neuroticism and depression; Wilson et al. (2007a) and Wilson et al. (2014); see more below] are not associated with neuropathological AD changes. Lastly, with the advent of specific therapies targeting amyloid plaques (Dyck et al., 2023), there is now the necessity of having biomarker evidence of abnormal amyloid (serum, cerebrospinal fluid, or PET) for the diagnosis of AD, which was not available in the published longitudinal loneliness studies. Thus, the present discussion, which attempts to understand the mechanisms underlying cognitive change due to loneliness, simply uses the more general term “dementia,” although the term could be “AD and related dementias” unless diagnosis includes neuropathology, since there is good evidence that the increased dementia among lonely individuals is not related to AD pathology (see below, section 3.3.1).

Though related to loneliness, social isolation is a more objective term that can be defined by number and type of social relationships. Loneliness is distinct from social isolation in that it is associated with a subjective sense of insufficient social connectedness. Of importance, social isolation is not consistently related to loneliness in many epidemiological studies. Humans are generally social animals needing interactions to maintain various aspects of health, but there are some people who opt to live alone without any negative feelings or outcomes related to loneliness. There are interpersonal differences in the need for both quantity and quality of social contact, e.g., trait differences such as those related to the introversion – extroversion dimension.

There are many theories and definitions of loneliness (Peplau and Perlman, 1982; Weeks, 1994). The definition we use here (i.e., the distressing feeling resulting from a perceived lack of social connection) aligns with many frameworks. Others have divided loneliness into subtypes, such as emotional or intimate loneliness (absence of meaningful relationships), social loneliness (perceived deficit in quality of social connections), and existential loneliness (feeling of fundamental separateness from others and the wider world) (Boss et al., 2015; Cacioppo et al., 2015c; Mansfield et al., 2021; Campaign to End Loneliness, 2023). The absence of a positive feeling of touch with conspecifics, i.e., positive thigmotaxis that is seen in many animal species, is likely one aspect of human loneliness.

There has been some attempt to better describe what social relationships (or their absence) contribute to loneliness (Rook, 1987). Lower quality of social contact is more related to loneliness than quantity (Pinquart and Sörensen, 2001). In any case, social isolation is not perfectly related to feelings of loneliness, with good evidence that the two are distinct, with independent impacts on cognition (Cardona and Andrés, 2023). For this reason, social isolation will not be a focus of this review.

There will also be no discussion of various societal causes of social isolation and loneliness, including attitudes toward race, ethnicity, religion, and gender. There is no discussion about loneliness: in people who already have dementia, possibly even as an early marker for dementia; in dementia care providers, or; in older adults secondary to societal ageism attitudes that contribute to a sense of loneliness. The specific focus of this paper deliberately limits the number of intervention studies discussed, since there are many studies targeting dementia caregivers that are of less clear relevance to the goal of this paper.

## 2 Background

### 2.1 Assessment of loneliness

The first psychometric assessment tool to assess loneliness was the 20-item Revised UCLA Loneliness Scale (Russell et al., 1980) that was later shortened to the 3-item loneliness scale (Hughes et al., 2004). Another psychometric scale consists of an 11-item full and 6-item reduced scale (De Jong Gierveld, 1987). Single-question Likert scales have often been used (Pinquart and Sörensen, 2001; Boss et al., 2015), e.g., a 4- or 5-point response to “how often do you feel lonely,” often dichotomized into two categories: lonely or not lonely. The single loneliness question is highly correlated to the multi-question scales (Pinquart and Sörensen, 2001). The other measure occasionally used is the single question about loneliness from the Center for Epidemiological Studies Depression (CES-D) scale (Radloff, 1977; Donovan et al., 2017). If that approach was used, then the researchers typically omitted that single CES-D loneliness question from the depression calculation. Direct questions may be better than the multi-question UCLA or De Jong-Gierveld scales if the goal is information on emotional aspects of loneliness or social interactions (Pinquart and Sörensen, 2001). Based on a meta-analysis, the UCLA loneliness scale is stable over a one-year interval, more so than the De Jong Gierveld scale, and has stability comparable to other personality traits (Mund et al., 2020).

Loneliness scales may be administered by a tester or self-administered, and there may be differences associated with age and sex depending on administration methodology (e.g., men may be less likely to admit to loneliness). It may be better if loneliness scales are self-administered in older adults. Loneliness has also been assessed via smartphone technology using ecological momentary assessment (Hammoud et al., 2021).

### 2.2 Epidemiology

Loneliness has been recognized as a health issue for some time (Peplau and Perlman, 1982; Weeks, 1994), with renewed prominence due to the COVID-19 pandemic (Hwang et al., 2020). Loneliness in the United States is considered an epidemic (eClinicalMedicine, 2023), with dire health consequences for mental and physical health, including cardiovascular disease and premature mortality. Loneliness is being increasingly recognized as a medical issue (Holt-Lunstad and Perissinotto, 2023), although there is still no medical diagnostic code. In the United Kingdom, perhaps more so than in the United States, loneliness is considered a significant public heath challenge, and the incidence of loneliness has not returned to pre-pandemic levels (National Academies of Sciences, Engineering, and Medicine, 2020; Campaign to End Loneliness, 2023; Department for Culture, Media, and Sport, 2023).

While most studies have evaluated non-Hispanic White samples, other racial groups also have a significantly increased likelihood of having worse cognition when experiencing loneliness (Wang et al., 2011; Chen et al., 2014; Zhou et al., 2018; Wilson et al., 2021).

#### 2.2.1 Chronicity

Transient feelings of loneliness are common, with 40% of adults over age 65 years reporting being lonely at least sometimes (Weeks, 1994; Pinquart and Sörensen, 2001). Chronic loneliness is less common than transient loneliness, but still common: 5–30% of older adults complain of frequent or chronic loneliness (Pinquart and Sörensen, 2001; Heinrich and Gullone, 2006; Theeke, 2009; Zhou et al., 2018; Chawla et al., 2021; Department for Culture, Media, and Sport, 2023). This range is generated mostly from high income countries, including one meta-analysis (Chawla et al., 2021).

#### 2.2.2 Education

An epidemiological study in China of over 5,600 rural older people, mostly low education and poor, observed a loneliness prevalence rate of 78.1% (Wang et al., 2011). Another study from China observed greater loneliness among rural and poorly educated individuals (Chen et al., 2014).

#### 2.2.3 Lifespan

While not consistently observed, the prevalence of loneliness varies slightly across the age span. In general, loneliness is most prevalent in adolescents before decreasing and remaining relatively stable throughout adulthood, with possible changes over age 80 (Pinquart and Sörensen, 2001; Shankar et al., 2013; Qualter et al., 2015; Luhmann and Hawkley, 2016; Hawkley et al., 2022; Department for Digital, Culture, Media, and Sport, 2023). While being a centenarian is unusual, it is not rare, and there are populations in the world with higher-than-expected prevalence of centenarians, e.g., Sardinians, Okinawans, and 7th day Adventists. Centenarians are less likely to report being lonely when compared to older populations with mean age 20 years less (Leitch et al., 2018).

#### 2.2.4 Gender

According to most studies, the prevalence of loneliness differs by gender, although there are exceptions (Chen et al., 2014). Greater incidence of loneliness is seen among women and is attributed to risk factors such as living arrangements, marital status, widowhood, and self-rated health (Chawla et al., 2021). An earlier paper supported this conclusion; while there was greater loneliness among women in marriage classes, there were no gender difference in non-married samples (single, widowed or divorced) (Pinquart and Sörensen, 2001; Campaign to End Loneliness, 2023). Of note, even though women have more loneliness, they may not have more social isolation (Shankar et al., 2013).

### 2.3 General health and mortality

Loneliness has a significant association with many factors that are related to worse cognition. Depression is one such factor, and is further described below. Loneliness also has a significant association with impaired sleep using multiple sleep measures (Cacioppo et al., 2002a; Jacobs et al., 2006; Hawkley and Cacioppo, 2010; Shankar, 2020; Benson et al., 2021). Loneliness is associated with obesity (Lauder et al., 2006), alcohol abuse (Akerlind and Hornquist, 1992), increased blood pressure (Hawkley et al., 2006, 2010), and addictive behaviors (Ingram et al., 2020). There are many aspects of general health, such as cardiovascular health and diabetes, that are associated with worse cognitive function and, additionally, there is often a decline in cognitive health prior to death.

An overview of 40 systematic reviews of loneliness and social isolation found they were associated with all-cause mortality, cardiovascular disease, and worse mental health outcomes across all ages (Leigh-Hunt et al., 2017). Social isolation and loneliness were also both associated with all-cause mortality in the English Longitudinal Study of Ageing; it was possible that some of the relationship between loneliness and mortality was due to social isolation (Steptoe et al., 2013), but that is not a consistent finding across other studies. A meta-analysis observed about a 30% increase in mortality from social isolation, loneliness, and living alone and, more specifically, a 26% increase in mortality due to loneliness after correction for multiple other mortality risk factors (Holt-Lunstad et al., 2015). The effects of social deficits were more predictive of death in samples with an average age younger than 65 years. Transient or situational loneliness increased mortality somewhat, but chronic loneliness trait had a greater effect than transient loneliness (Shiovitz-Ezra and Ayalon, 2010).

## 3 Cognitive function

There is a large literature evaluating the relationship between cognitive function and loneliness. Some of these studies have evaluated younger adults, and there even exist some experimental studies of loneliness in humans and non-human animals (Cacioppo et al., 2000; Cacioppo and Hawkley, 2009; Cacioppo et al., 2015b; Vitale and Smith, 2022). However, because this review is focused on non-experimental loneliness studies in older adults, most of this literature is not discussed. Even with this constraint, there are still many cross-sectional and longitudinal studies evaluating cognition associated with loneliness, resulting in several systematic reviews and meta-analyses (Boss et al., 2015; Kuiper et al., 2015; Penninkilampi et al., 2018; Lara et al., 2019; Harrington et al., 2023). However, these review articles all have significant limitations, given the weaknesses of the cross-sectional studies in this field. To best understand the relationship between loneliness and cognitive decline in the present review, we focus on the relationships between loneliness and cognitive change by: (1) reporting on general cognitive decline associated with loneliness in longitudinal studies, not simply cross-sectional studies; (2) describing the specific cognitive domains associated with loneliness; (3) describing dementia-related biomarkers, including neuropathology, associated with loneliness; and (4) highlighting known confounds of cognitive decline that also relate to loneliness (depression, being the most problematic).

### 3.1 Cognitive function decline and loneliness

There is a significant increase in the likelihood of dementia among people experiencing loneliness. Almost all cross-sectional and longitudinal studies that have evaluated loneliness have found a relationship between loneliness and measures of global cognitive function (see review papers Boss et al., 2015; Kuiper et al., 2015; Penninkilampi et al., 2018; Lara et al., 2019; Harrington et al., 2023; and see Table 1 for all longitudinal studies and see end of this section for discussion of HR’s for development of dementia). In the case of loneliness and its association with cognitive function, it is important to control for potentially confounding dementia risk factors such as age, years of education, physical activity, vascular risk factors, and socioeconomic status (SES). While there are many potential risk factors for development of dementia (Barnes et al., 2012; Livingston et al., 2020), most such as diabetes are not related to loneliness. Depression is of particular concern, since depression is associated with loneliness, some measures of depression are overtly confounded by loneliness, and depression increases the risk of dementia.

**Table 1:** Longitudinal studies of loneliness and cognition.

Study	N (female)	Age in years at study start (± SD)	Region	Loneliness assessment	Screening	Visit schedule (design + stability of baseline)	Incidence of dementia (or comparable measure)	Loneliness hazard ratio (HR; OR; RR)	Biomarkers and dementia pathology	Patterns of cognitive changes related to loneliness	Depression	Notes
Tilvis et al. (2004)	650 (73.0%)	Split sample of 75- (239), 80- (212), and 85-year-olds (199)	Helsinki, Finland	Likert (single-item, “Do you suffer from loneliness?”)	-	Single baseline assessment, with 1-, 5-, and 10-year follow-ups	Cognitive impairment (MMSE <24) apparent at baseline for 29.6 and 38.6% of men and women, respectively; 44% of baseline impaired group showed cog decline (drop of ≥4 for MMSE) at 10-year follow-up, compared to 34.1% of baseline non-impaired, but 60% of impaired had CDR class >0.5 at endpoint, compared to 20% of non-impaired	Cognitive decline RR = 3.0 at 10-year follow-up	APOE4; blood plasma lipids and glycemic data; ionized serum calcium; C-reactive protein (CRP); dementia type and presence determined by neurologist at baseline	Cognitive decline (MMSE; CDR) associated with feelings of loneliness at the 10-year follow-up	Measured depression (single-item yes/no, “Do you feel yourself depressed?”), but did not control for depression in analysis	-
Bennett et al. (2006)	89 (55.1%)	84.3 ± 5.6	Chicago, United States	Interview about social network size (# children, # family, and how often they interact)	Review of clinical assessment results by neuropsychologist prior to inclusion in the study to determine presence of dementia	Single baseline with annual clinical follow-up assessments until death and post-mortem autopsy	Global AD pathology proximate to death = 0.70	-	Amyloid load; Tau tangles; AD pathology global measure based on modified Bielschowsky silver stain	Social networks not related to global AD pathology, but identified as possible protective factor (i.e., global cognition better at all levels of dementia pathology with increased social network size), and effects most pronounced for semantic memory (Boston Naming, verbal fluency, and reading test) and working memory (digit span for- and backward, and digit ordering), interaction strongest for neurofibrillary tangle pathology and episodic memory, but most striking for verbal memory; effect for episodic memory (story recall) and tau tangles, but not perceptual speed (symbol-digit modalities; Stroop; number comparison), or visuospatial ability (line orientation; Raven’s matrices)	Social network not related to depression (CES-D, not excluding loneliness question)	-
Wilson et al. (2007b)	791 (75.7%)	80.7 ± 7.1	Chicago, United States	Modified De Jong-Gierveld Loneliness Scale (5-item; Likert)	Absence of clinical diagnosis of dementia was required for enrollment	Single baseline and annual follow-up until death (range of 2–5 total evaluations; mean 3.3)	76 (9.6%) participants developed clinical AD or possible AD over the course of follow-up	Clinical AD RR = 1.51 (risk of AD more than doubled in lonely 90^th^ % compared with not-lonely 10^th^ %)	Uniform clinical evaluation to detect dementia and AD; post-mortem brain analysis of amyloid burden, tau tangles, Bielschowsky silver stain, and cerebral infarction	Loneliness unrelated to global AD pathology or cerebral infarction; loneliness associated accelerated decline in global cognition, semantic memory (verbal fluency; BNT; NART), perceptual speed (number comparison; Symbol Digit Modalities Test; modified Stroop), and visuospatial ability (Judgment of line orientation; standard progressive matrices) across follow-up, but not episodic memory (story recall) or working memory (Digit Span front-, backwards, and ordering)	Controlling for depression (CES-D, excluding loneliness question) reduced association of loneliness with AD risk by 16% (RR = 1.41); controlling for loneliness reduced association between depression and AD risk (RR = 1.06); reports of loneliness in excluded CES-D question linked to increase of 86% in AD risk	-
Chen et al. (2011)	1,526	≥ 65	China	Social isolation assessed through review of general health and risk factors records	Required participants to have at least minimal education and excluded individuals who were either <65 years of age or who had dementia at baseline	Single baseline, 1-year follow-up, plus additional follow-up up to 7.5 years post-baseline	14.7 persons per 1,000 (age-standardized)	Incident dementia OR = 1.00 when living alone, versus OR = 0.36 when living with spouse, (grand )-children, or parents	No physiologic measures; computer-assisted AGECAT approach to classify Geriatric Mental State (GMS) Questionnaire data and included diagnostic information from psychiatrists	Increased incidence of dementia for individuals living with fewer family members	Did not examine relationship between incident dementia and baseline depression, but reported possible relationship between depressive symptoms and dementia	Did not report % female; unclear description of dementia diagnosis; did not specify measurement of depressive symptoms; unclear if all of sample made it through follow-up
Ellwardt et al. (2013)	2,255 (54%)	63 ± 6.65	Netherlands	De Jong-Gierveld Loneliness Scale (11-item)	Aged 55–85 years; excluded individuals without signs of dementia at baseline, who had MMSE ≥24; only included participants with complete data for control variables	Single baseline and two follow-up assessments: every 3 years, for a total of 6 years	Did not report	-	-	Reduced feelings of loneliness mediated the relationship between higher levels of emotional support and measures of cognitive functioning (MMSE; Coding task; raven colored progressive matrices)	-	Reverse causality addressed with cross-domain latent growth models
Shankar et al. (2013)	6,034 (54.7%)	65.6 ± 9.5	England	Short form UCLA Loneliness Scale (Likert; 3-item)	Participants over 50 years of age	Single baseline and follow-up after 4 years	Did not screen for dementia	-	-	Higher loneliness associated with poorer verbal fluency (category naming), immediate recall (word list), and delayed recall at baseline; loneliness associated with decreases in immediate and delayed recall at follow-up, but not verbal fluency	Depression (CES-D, excluding loneliness question) positively associated with loneliness; CES-D included as covariate, but did not change results	-
Holwerda et al. (2014)	2,173 (63.1%)	65–86	Amsterdam, Netherlands	Single yes/no question: “Do you feel lonely or do you feel very lonely?”	Excluded participants with dementia at baseline	Baseline and 3-year follow-up (median = 38 months)	13.4% of 433 participants with baseline feelings of loneliness developed dementia at follow-up, compared to only 5.7% of the 1,740 individuals with no baseline loneliness	Dementia OR = 1.64 at follow-up in the multi-variate analysis	No physiologic measures; GMS-AGECAT scores to determine presence of dementia	Increased loneliness at baseline (but not social isolation) was related to increased risk of dementia at follow-up (multi-variate analysis)	Controlled for depression (GMS-AGECAT); no interaction between feelings of loneliness and depression	-
Zhong et al. (2016)	2,995 (50.6%)	75.6 ± 8.3	China	Likert; single-item (“How often do you feel lonely?”)	Age 65 and older; excluded respondent with missing visit data; excluded individuals with cognitive impairment at baseline (mMMSE <14)	Single baseline and two follow-up assessments: every 3 years, for a total of 6 years	Did not report	-	-	Transient and chronic loneliness both significantly associated with lower cognitive function (modified-MMSE; mMMSE) after 6 years of follow-up	-	-
Donovan et al. (2017)	8,030 (60.0%; based on inflated baseline sample of 8,382)	73.2 ± 6.47	United States	Likert (single item from CES-D)	Over age 63 years (65 years at first follow-up); removed individuals from analysis who had incomplete data	Single baseline with biennial assessments (1998–2010)	Did not report	Low cognition predicted loneliness (OR = 1.3), but was no longer significant after controlling for baseline depression (OR = 1.1)	-	Baseline loneliness predictive of cognitive decline (memory from m-TICS word list, or proxy rater score on IQCODE), but effect was only marginally significant when adjusted for depression interacting with time; 20% greater cognitive decline over 10 years for lonely compared to non-lonely and depressed compared to non-depressed; only assessed episodic memory (TICS 10-word list)	Depression (CES-D excluding loneliness question) as covariate weakened relationship between baseline loneliness and decrease in cognition; cognition at baseline predicted loneliness over time, but not after controlling for depression	Some individuals had impaired cognition at baseline and were still included
Zhou et al. (2018)	7,867 (54.9%)	83.09 ± 10.92	China	Likert; single-item (“Do you feel lonely?”)	Excluded participants under age 65 at baseline, and who had dementia at baseline	Single baseline and 3-year follow-up (included participants who suffered from dementia at time of death before follow-up)	393 out of 7,867 participants developed dementia by follow-up assessment defined as “yes” response to questions “Are you suffering from dementia?” and “Have you been diagnosed with dementia by a physician?”	Dementia OR = 1.31	-	Loneliness was related to increased risk of dementia before and after controlling for lifestyle and baseline health; significant gender interaction, such that loneliness increased dementia risk more for men than women	-	-
(Yin et al., 2019)	5,885 (55.4%)	65.3 ± 9.0	England	Short form UCLA Loneliness Scale (Likert; 3-item)	Age 50 and older; excluded individuals with diagnosis of stroke or dementia at baseline and stopped participation when diagnosis developed during follow-up	Single baseline with follow-up every 2 years, up to 10 years	-	-	No physiologic measures; dementia determined self- and informant-reported clinical diagnosis, or informant-reported IQCODE score > 3.38	Increased baseline loneliness associated with worse memory (word recall) and semantic verbal fluency (animal naming task) at baseline, as well as decline of memory and verbal fluency across follow-up visits	Covaried for depression (CES-D, minus question on loneliness)	-
Griffin et al. (2020a)	6,654 (59.0%)	72.57	United States	Short form UCLA Loneliness Scale (Likert; 3-item)	Age 65 and older; final analysis used only individuals with complete cognitive assessment data	Single baseline with follow-up every 2 years, up to 6 years	242 individuals with cognitive impairment included at baseline; incidence across follow-up not reported	-	mTICS ≤8 used to determine cognitive impairment	Greater loneliness predicted lower cognitive function (mTICS), but not change in cognitive function	CES-D (8-item) included as covariate (not excluding loneliness question)	-
Kuiper et al. (2020)	378 (66.1%)	70.7 ± 7.4	Netherlands	De Jong-Gierveld Loneliness Scale (11-item)	Included participants over 60 years of age, with a primary diagnosis of major/minor depression or dysthymia; excluded participants if they had dementia, severe psychiatric disorder other than depression, or MMSE <18	Single baseline and follow-up at 2-years	No participants had dementia at baseline; did not report or screen for dementia at follow-up	-	-	Measured processing speed (Stroop), interference control (Stroop), verbal memory (modified auditory verbal learning test), and working memory (WAIS digit span); loneliness only associated with impaired working memory, but no longer significant after controlling for covariates	Study was conducted using a sample of adults with DSM-IV current depressive disorder; controlling for severity weakened relationship between baseline loneliness and decline in working memory	-
McHugh Power et al. (2020)	7,433 (53.36%)	63.99 ± 9.83	Ireland	UCLA Loneliness Scale (Likert; 5-item; dropped item #4 from models due to poor factor loading)	Over age 50 years; excluded individuals who self-reported having physician-identified AD, dementia, organic brain syndrome, senility, serious memory impairment, or other serious psychiatric or emotional problems	Single baseline and follow-up at 2 and 4 years	Did not report	-	-	Higher loneliness at baseline was predictive of worse cognitive functioning at 4-year follow-up (aggregate of immediate recall, delayed recall, MMSE, and verbal fluency)	Depressive symptoms (CES-D 20-item; dropped items #4, 15, and 19 due to poor factor loading) partly mediated the relationship between loneliness and cognition	-
Rafnsson et al. (2020)	6,677 (55.7%)	66.0 ± 9.4	England	UCLA Loneliness Scale (Likert; 3-item)	Over age 50 years; excluded those with dementia at baseline (physician diagnosis or short-form IQCODE ≥3.5)	Single baseline, plus 4 follow-up assessments, every 2-years (note: baseline visit was visit #2 of parent data set, which is when loneliness was first assessed)	220 out of 6,677 participants developed dementia during follow-up	Multivariate dementia HR = 1.44, and HR = 1.33 with additional social interaction variables	-	Loneliness positively correlated with risk of dementia in both uni- and multi-variable analyses, but no such correlation between social isolation and risk of dementia	Depression added as covariate (CES-D, excluding loneliness questions), but loneliness remained significant	Addressed reverse causality by excluding cases diagnosed within 24 or 48 months of baseline and results did not change; use of “enhanced” dementia determination from MMSE and word list recall scores did not change results
Sundstrom et al. (2020)	1,905 (52.6% for no-dementia; 65.0% for all-cause dementia)	71.5 ± 9.3 (no dementia); 74.7 ± 7.6 (all-cause dementia)	Sweden	Single yes/no question (“Do you often feel lonely?”)	Age 60 and older at baseline; excluded individuals with missing dementia status data or insufficient follow-up period	Single baseline (waves 3 or 4 of parent study), plus variable number of follow-ups every ~5 years, up to 20 years (mean of 11.1 years)	428 individuals developed dementia during follow-up (221 AD; 157 VaD; 50 other)	All-cause dementia HR = 1.46; AD HR = 1.69; VaD HR = 1.34	Variable process to determine dementia status and subtype (chart reviews, which may or may not have included imaging)	Baseline loneliness positively associated with increased risk of all-cause dementia, specifically AD (as labeled by the study), but not vascular dementia	Depression added as covariate (CES-D, minus question about loneliness), but did not affect significance of loneliness in predicting dementia	Addressed reverse causality by rerunning analysis and removing cases of dementia onset in first 5 years of follow-up (results stayed the same); unclear if AD label in study overall was appropriate since there were insufficient pathologic markers
Sutin et al. (2020)	12,030 (60.0%)	67.30 ± 10.45	United States	UCLA Loneliness Scale (Likert; 3-item)	Age 50 and older, without dementia at baseline; excluded participants without at least a single follow-up assessment	Single baseline and follow-up assessments every 2 years for 10 years	1,104 (9%) participants developed dementia (defined as TICS-m ≤ 6; composite of word recall, serial subtraction, and backward counting) over the course of follow-up	Dementia HR = 1.40	APOE4 status added as covariate when available for subset of sample (n = 9,775)	Each point increase in loneliness associated with 40% increase in dementia risk (TICS-m), both before and after controlling for behavioral risk factors, social isolation, and depressive symptoms	Depression added as covariate (CES-D, minus question about loneliness), but loneliness still significant (HR = 1.19)	Sensitivity analysis confirmed that loneliness still related to increased dementia risk when excluding individuals w/ CIND at baseline (HR = 1.44)
Windsor et al. (2020)	516 (50.0%)	84.9	Berlin, Germany	UCLA Loneliness Scale (Likert; 8-item)	Age 70 and older; excluded data points in cases where MMSE scores indicated probable dementia	Single baseline, with 5 follow-ups across 13 years	148 participants classified as having probable dementia at baseline; 258 out of 905 assessments across all follow-ups fell below the MMSE cutoffs for probable dementia	-	Dementia status determined by short MMSE at each wave of assessment (age cohort-specific cutoffs)	Perceptual speed (digit-letter test) and category fluency (animal naming) were not reliably associated with baseline loneliness	Controlled for depressive symptoms (German version of CES-D)	Addressed reverse causality by looking at subset (*n* = 368) of sample without dementia, and also by adding wave-specific probable dementia status as a covariate for the full sample
Akhter-Khan et al. (2021)	2,880 (53.9%)	62.1 ± 9.0	United States	Likert (CES-D single-item)	Age 45 and older who had no dementia at baseline; excluded participants with missing follow-up or dementia data	Exam #7 of parent study treated as single baseline, with variable multiple repeated follow-ups on average every 4 years, up to a maximum of 18 years	218 individuals (7.6%) developed dementia and AD (as labeled by the study)	Dementia HR = 1.91 (persistent loneliness); transient loneliness evinced lower risk of dementia (HR = 0.34) than no loneliness	APOE4 status added as covariate	Individuals with transient loneliness had decreased risk of developing dementia, relative to no loneliness, while persistently lonely individuals had an increased risk of developing dementia	Split sample depressed vs. not depressed (CES-D 20-item version; excluding loneliness question); loneliness effects not significant in depressed cohort	-
Freak-Poli et al. (2022)	4,514 (study 1, 57.0%); 2,112 (study 2, 36.0%)	71 ± 7 (study 1); 72 ± 10 (study 2)	Rotterdam, Netherlands (study 1); Stockholm, Sweden (study 2)	Likert (CES-D; study 1); Binary yes/no (study 2)	Age ≥ 55 (study 1) or ≥ 60 (study 2); excluded if MMSE <25 (study 1) or < 26 (study 2), or if individual had major depression or dementia	Single baseline, with multiple repeating follow-ups, every 4–5 years, up to 14 years (study 1), or every 3 or 6 years, up to 10 years (study 2)	-	Dementia HR = 1.34 (study 1) and HR = 2.16 (study 2)	-	Increased baseline loneliness associated with decline in MMSE scores and increased dementia risk in both studies	Controlling for depression (CES-D in study 1; CPRS in study 2) did not change results	Sensitivity analysis addressed reverse causality by excluding first 5 years of follow-up – only slight decrease in effect sizes, though study #2 loneliness effect was no longer significant (*p* = 0.06)
Salinas et al. (2022)	2,308 (dementia test sample, 56.0%); 1,875 (cognition test sample, 54.0%)	73 ± 9 (dementia test sample); 62 ± 9 (cognition test sample)	United States	CES-D (Single-item; Likert)	Age 60 and older, with no dementia at baseline and no missing dementia data at follow-up	Single baseline with follow-up monitoring for 10-year period (dementia sample only)	329 (14%) of dementia test sample developed dementia during follow-up	Dementia HR = 1.54 in overall sample	APOE4; MRI (brain matter volume; white matter injury); monitoring of MMSE scores	Measured memory (logical memory delayed recall), executive function 9trails making test, and global cognition (trails making test, logical memory, visual reproductions, paired associate learning, Hooper visual organization test, and similarities test); increased baseline loneliness associated with higher 10-year dementia risk, poorer executive function, and increases in atrophy/injury markers	Controlling for depression eliminated relationship between cognition and loneliness in the cognition test sample	-
Jackson et al. (2023)	810 (70.1%)	82.8 ± 6.2	Chicago, United States	De Jong-Gierveld Loneliness Scale (modified 5-item Likert version)	Participants age 65+ with no known dementia at baseline	Collapsed two studies (study 1, MAP; study 2, MARS); single baseline plus yearly follow-up evaluations for up to 20 years (study 1) or 12 years (study 2)	Did not report	-	Post-mortem pathology, including amyloid burden, tau tangles, gross chronic cerebral infarctions, chronic microinfarcts, Lewy body disease, TPD-43, hippocampal sclerosis, cerebral amyloid angiopathy, cerebral atherosclerosis, and arteriolosclerosis; APOE4 covariate	Baseline loneliness was inversely related to measures of global cognitive resilience (composites of 19 tests, spanning episodic memory, working memory, semantic memory, perceptual speed, and visuospatial ability/perceptual orientation domains), and this relationship held before and after accounting for social isolation; change in loneliness was also inversely related to change in cognitive resilience, but not the final time point in the fully adjusted model	-	-
Sutin et al. (2023)	492,322 (54.5%)	56.55 ± 8.09	England	Single yes/no question (“Do you often feel lonely?”)	Excluded cases where individuals had ICD codes or self-reported acknowledgment of dementia prior to baseline	Single baseline and variable follow-up over 15 years	7,475 (1.5%) participants developed all-cause dementia	All-cause dementia HR = 1.59	APOE4 status as covariate	Baseline loneliness associated with ~60% increase in all-dementia risk	Depression (PHQ-2) somewhat attenuated but did not eliminate the relationship between loneliness and all-cause dementia when added as a covariate	-
Yu and Siang Ng (2023)	7,037 (57.4%)	67.97 ± 9.65	United States	UCLA Loneliness Scale (Likert; 3-item)	Age ≥ 50 years; excluded individuals with dementia at baseline	Single baseline and follow-up after 4 and 8 years	200 (3%) and 252 (4.45%) participants had developed dementia in the 4- and 8-year follow-ups, respectively	OR’s ranged 0.80–0.82 (odds of not having cognitive decline)	Hemoglobin A1C; LDL and HDL Cholesterol; C-reactive protein; Cystatin C	More loneliness associated with worse cognitive status (TICS-m) in follow-up, but not in model with all covariates; HbA1C was only biomarker to mediate relationship between cognitive status and loneliness	Depression added as covariate (CES-D excluding loneliness question); loneliness no longer significant, but maybe due to multiple health variables added along with depression	-

The relationship between loneliness and cognition is not dependent on the method of loneliness assessment, since a significant association between the two has been demonstrated using the de Jong Gierveld scale (Wilson et al., 2007b), the 3-item UCLA scale (Shankar et al., 2013; Sutin et al., 2020), the single CES-D loneliness question (Donovan et al., 2017; Akhter-Khan et al., 2021), or a simple yes/no question (Sundstrom et al., 2020). One study directly compared the CES-D single question to the 3-iterm UCLA scale and found no difference between the assessments regarding the effect of loneliness on cognition (Sutin et al., 2020).

An important limitation of cross-sectional epidemiological studies is that early AD brain changes may produce behavioral symptoms of dementia in people who still appear clinically normal on screening tests, resulting in reverse causality. More specifically, people with brain changes in early dementia who are likely to develop symptomatic dementia in the near future may intentionally decrease their social interactions, perhaps because of cognitive processing limitations that make social interactions less positive. As a result, some longitudinal studies looking at loneliness have taken care to analyze subgroups that started study participation as cognitively normal and who were followed for 2 or more years without obvious cognitive change [e.g., Rafnsson et al. (2020) and see Table 1 that only has longitudinal studies]. We cite some of the relevant, larger, better characterized or unique cross-sectional studies for completeness, but do not feel they can shed sufficient light on the underlying cause of the relationship between loneliness and cognitive decline.

Table 1 gives more details of relevant longitudinal studies, so only more critical points are discussed here. In one of the earlier longitudinal studies, older adults were assessed for loneliness at baseline and were then followed until death (Wilson et al., 2007b). The risk of dementia was more than doubled in lonely persons (those who scored in the 90th percentile of loneliness compared with those scoring in the 10^th^ percentile). The relative risk increase for each loneliness scale point was 1.51. Another earlier longitudinal study from Finland found that at baseline, 17.6% reported feeling loneliness much of the time (Tilvis et al., 2004). At one- and five-year follow-ups, baseline general health issues had the greatest effect on cognitive decline as assessed with Mini-Mental State Exam (MMSE) and Clinical Dementia Rating scale. These health factors included claudication, diabetes, hypertension, and stroke history. Of note, Finland has a higher prevalence of vascular risk factors than other countries, perhaps related to more smoking and salt intake, among others; thus, the sample from this region likely has a higher incidence of vascular dementia. The loneliness effect on cognitive decline at 10 years was significant, as was the APOE4 effect, but not hypertension or diabetes. There was one study demonstrating an effect of loneliness on cognition at age 70 that had access to some cognitive tests at age 11 from a much older childhood study, but it was essentially a cross-sectional study at age 70 (Gow et al., 2013). The Amsterdam study of elderly observed data from over 2,000 individuals and measured an odds ratio (OR) to develop dementia over 3 years of 1.64 after adjustments in the final model; the relationship with loneliness was mostly independent of depression (Holwerda et al., 2014).

Donovan observed a 20% increase in cognitive decline over 10 years in lonely individuals compared to non-lonely, as well as in those depressed at baseline compared to not depressed (Donovan et al., 2017). Poorer cognition at baseline predicted greater loneliness over time but not after adjusting for baseline depression. Roughly half of lonely people endorsed depression. Cognition did not predict changes in loneliness. This same study evaluated socio-demographic risk factors (i.e., advanced age, female sex, low education, low SES) as modifiers of the relationship between loneliness and cognitive decline and found that none of them contributed significantly to the relationship between loneliness and cognition (Donovan et al., 2017).

A study in China observed increased dementia risk related to loneliness at 3-year follow-up with an OR of 1.31 (Zhou et al., 2018). Additionally, men who felt lonely were more likely to develop dementia than women, which is an inversion of the pattern reported in most studies. A health and retirement study observed that each point increase in loneliness scale increased risk of dementia by 40%, with a hazard ratio (HR) of 1.41 over 10-year follow-up (Sutin et al., 2020). Importantly, to deal with reverse causality, this study also analyzed subgroups with 6 years follow-up or excluding those with cognitive impairment not demented (CIND), and this approach did not significantly change the loneliness risk.

A Framingham study using the single loneliness item from the CES-D allowed for an 18-year follow-up from a midlife measure of loneliness (Akhter-Khan et al., 2021) with a later DSM-4 diagnosis of dementia. Persistent loneliness, which was observed in 8.8% of the sample, increased the risk of dementia 13.4% vs. 7.5% (HR 1.91, 1.25–2.90). Also, transient loneliness is intrinsically different than persistent loneliness and was unexpectedly associated with a decreased risk of dementia. This observation of transient loneliness having the opposite effect of persistent loneliness on cognition was also noticed in a different longitudinal study in China (Zhong et al., 2016). Another Framingham population-based study observed that loneliness was associated with a higher 10-year dementia incidence rate. However, this dementia rate among the lonely was not different for those over age 80 years, but being lonely and less than 80 had twice the dementia risk (Salinas et al., 2022).

A 20-year study in Sweden observed that loneliness increased the likelihood of all-cause dementia (Sundstrom et al., 2020). That study judiciously excluded patients with onset of dementia within 5 years of baseline to avoid reverse causality. They still observed that the loneliness effect was significant with an increased likelihood for all-cause dementia (HR 1.46), for what they called AD (HR 1.69), but not for vascular dementia (HR 1.34). The paper stated that multiple specialists contributed to the diagnosis, but there was no citation to specific criteria or to other dementia diagnoses such as Lewy body disease or fronto-temporal dementia.

In an extremely large UK Biobank Study (*n* = 492,322), feeling lonely was associated with a nearly 60% increase in all-cause dementia (HR 1.59), with loneliness being a stronger predictor of vascular dementia than what they called AD. This increased risk was at least partly independent of depression, social isolation, clinical syndromes (e.g., diabetes) and behavioral factors (e.g., physical activity) (Sutin et al., 2023).

One paper analyzed data from two longitudinal samples: the Rotterdam study (*n* = 4,514) and the Swedish National study (*n* = 2,112) (Freak-Poli et al., 2022). At baseline participants were free of major depression and had MSSE score > =26. Loneliness was prospectively associated with decline in MMSE in both cohorts. Additionally, both had increased risk for developing dementia (HR 1.34). Adjustment for depression and exclusion of the first 5 years of follow-up did not alter the results of the analysis.

This section has highlighted the relatively strong relationship between loneliness and decline in cognitive function in longitudinal studies. The HR for development of dementia in longitudinal studies (Table 1) ranged from 1.34 to 2.16 with an arithmetic mean of 1.61. The total number of participants followed longitudinally until development of dementia was over 500,000 and the simple arithmetic mean HR for the studies that provided it (1.61) was remarkably close to the HR from the largest longitudinal study, 1.59 (Sutin et al., 2023). This relationship may be stronger among younger individuals. As well, this relationship has held up even when accounting for the reverse causality, or the potential for early brain changes to produce behavioral symptoms such as loneliness even before cognitive decline has occurred. The better studies have used a follow-up period of at least 2 years without clinical cognitive decline to deal with this issue, and one fortunate study had an 18-year follow-up after enrollment without cognitive decline. This reinforces the notion that cross-sectional studies looking at the relationship of loneliness to incidence of cognitive decline are problematic. In general, adjustments for other risk factors for cognitive decline such as depression, physical activity, and even social isolation have not consistently altered the relationship of cognitive decline to loneliness.

### 3.2 Pattern of cognitive deficits

Further understanding of the underlying mechanism of how loneliness produces cognitive changes requires evaluation of the pattern of those cognitive changes. In younger adults, it has been stated that effortful attentional processes may be impaired by loneliness (Cacioppo et al., 2000). In older adults, many studies simply used a screening test such as the MMSE to assess global cognitive function. While this approach helps to detect global cognitive changes, it does not shed light on the possible underlying causes that produce changes in different cognitive domains. Unfortunately, most studies in older adults did not use more than one cognitive assessment measure, e.g., demonstrating decline on a 10-word list learning test (Donovan et al., 2017). In this vein, a systematic review and meta-analysis of loneliness and cognitive function in older adults without dementia demonstrated that there was an impact on global cognition in the meta-analysis, but the data were too heterogeneous to sort out effects in specific cognitive domains (Harrington et al., 2023). Part of this lack of effect may be related to how most of the studies in the meta-analysis were cross-sectional, resulting in the problems of interpretation mentioned previously, exacerbated by the limitations of not often using a standard broad cognitive battery.

In a longitudinal study of 800 older non-demented adults, loneliness was associated with cognitive decline (Wilson et al., 2007b). The interaction of loneliness with cognitive decline over time was significant for global cognition, semantic memory, perceptual speed and visuospatial ability (all interaction *p* values less than 0.05), but, interestingly, not for episodic memory (*p* = 0.79). The loneliness effect on working memory decline was marginally significant (*p* = 0.09). In one large cross-sectional study, 13,176 adults over age 65 years who did not self-report AD or dementia completed a 4-domain cognitive assessment: immediate recall of a 15-word list, delayed recall of the word list, semantic category fluency and a timed Mental Alternation Task (Gilmour, 2011). Those who were lonely scored in the 30th percentile on the immediate recall, semantic fluency and processing speed. Poor performance on delayed recall, which is an early marker for AD brain changes, was not significantly associated with loneliness.

In the English Longitudinal Study of Ageing people over age 50 (*n* = 6,034), participants were assessed at baseline using a cognitive battery (immediate and delayed recall of a 10-word list and category verbal fluency), and again 4 years later (Shankar et al., 2013). At baseline, loneliness was significantly associated with all three cognitive measures. At 4-year follow-up, after adjusting for baseline cognitive function, loneliness was associated with declines in immediate and delayed recall, but no longer with verbal fluency. This study was interested in social isolation, so loneliness and social isolation were entered at the same step in the regression analysis and consequently limited interpretation of the loneliness effect. There was an interesting interaction with educational level, such that only those with low levels of education had poorer memory with loneliness. A later analysis of the English Longitudinal Study of Ageing focused on bidirectional associations between loneliness and cognitive functioning; this analysis did observe that loneliness was associated with decline in performance on both a 10-word list memory task and a category verbal fluency task over 10 years (Yin et al., 2019).

A birth cohort study had some cognitive testing at age 11 but was essentially a cross-sectional study at age 70 (*n* = 1,091) (Gow et al., 2013). Principal component analysis measures were calculated from a fairly extensive cognitive battery: general cognitive ability, processing speed, and memory. Loneliness was associated with the three component domains even after adjusting for an age 11 IQ score.

In the Berlin Aging Study, loneliness at baseline was not reliably associated with cognition as assessed by perceptual speed (Digit Letter test) or category fluency (Windsor et al., 2020). In the Longitudinal Aging Study of Amsterdam, cognition was assessed with MMSE, a perceptual speed Coding Test, and Raven’s Matrices (Ellwardt et al., 2013). Reduced feelings of loneliness appeared to indirectly mediate the relationship between higher levels of emotional support on cognition.

In an analysis of a subset from the Framingham study who had a cognitive assessment battery (*n* = 1875), loneliness as assessed with the CES-D question was associated with poorer cognition in the executive function domain (Salinas et al., 2022). Despite loneliness being associated with a higher 10-year dementia incidence rate, there was no significant association between loneliness and global cognitive score, Logical Memory Delayed Recall, or hippocampal volume.

The above noted systematic review (Harrington et al., 2023) had seven longitudinal studies with several cognitive measures. The systematic review did not have enough information on cognitive domains for the meta-analysis but did note in the narrative review that there was an association of loneliness with worse global cognition, episodic memory, working memory, visuo-spatial function, processing speed, and verbal fluency. However, it needs to be reiterated that most of the included studies were cross-sectional (Harrington et al., 2023).

This section has highlighted the inconsistency of the domains of cognitive function that have been was assessed as well as the specific cognitive tests. Focusing on the domain of episodic memory, most studies have not shown declines in this domain highly associated with AD pathology even while showing changes in other domains. However, there are a couple of studies that have shown a decline.

### 3.3 Dementia biomarkers associated with cognitive decline and loneliness

#### 3.3.1 Neuropathology

Some studies have looked at the underlying neuropathology of loneliness and cognitive decline. While there was a relationship between loneliness and dementia incidence in a longitudinal study, there was no relationship between loneliness and the neuropathological markers of AD pathology or cerebral infarction (Wilson et al., 2007b). Another study also found a relationship between loneliness and dementia, but no relationship between loneliness and AD pathology (Jackson et al., 2023). This latter study found that, in some sense, loneliness decreased cognitive reserve, i.e., the amount of AD neuropathology observed to produce dementia in people with loneliness was significantly less than in those without loneliness. There are useful papers for further discussion of cognitive reserve as some property such as higher education or occupational attainment that decreases the likelihood of development of dementia or AD (Stern, 2012; Pettigrew and Soldan, 2019). This trend may be similar to the observation of increased neuroticism relating to increased incidence of dementia but not increased AD neuropathology (Wilson et al., 2007a). Two cross-sectional papers from another group suggested a relationship between loneliness and amyloid (Donovan et al., 2016) or tau (d'Oleire Uquillas et al., 2018). However, biomarkers of loneliness in cross-sectional studies in the absence of longitudinal data make it impossible to know whether reverse causality may explain these relationships. The more robust longitudinal studies specifically address this issue by analyzing people who have been followed at least 2 years with no cognitive changes.

#### 3.3.2 APOE4, loneliness, and cognitive decline

APOE4 is a known risk factor associated with AD dementia and pathology; a single copy of APOE4 produced a HR of 1.75 for development of MCI/dementia across four large studies with a total of almost 17,000 participants followed longitudinally (Qian et al., 2017). The significantly increased risk of dementia with loneliness in a very large study from the U.K (*n* = 490,000) held after correction for APOE4. The loneliness risk was seen in both APOE4 carriers and non-carriers, but was stronger among non-carriers (Sutin et al., 2023). An earlier study also found the increased dementia risk for loneliness held after correction for APOE4 as well social isolation (Sutin et al., 2020). The relationship of APOE4 to the development of dementia in the presence of loneliness may be dependent on age. In another study, the HR for 10-year dementia incidence was 1.54 (Salinas et al., 2022). However, there was no clear association between APOE4 and dementia for those over age 80, while lonely participants under 80 without APOE4 alleles had a much higher dementia risk with a HR of 3.03 (Sutin et al., 2023).

#### 3.3.3 Neurobiology and neuroimaging

To our knowledge there has been one systematic review of the neurobiology of loneliness (Lam et al., 2021), but most papers surveyed in this report included young adults. In a large group of younger adults, loneliness was associated with greater regional grey matter volume in the dorsolateral prefrontal cortex, which was hypothesized to be attributed to immature emotional regulation, and this relationship was partially mediated by neuroticism (Kong et al., 2015).

In older adults, individuals with higher loneliness scores had smaller gray matter volumes in the left amygdala/anterior hippocampus, the left posterior hippocampus, and the left cerebellum (Duzel et al., 2019). A very large but exploratory study of 10,000 40–69 year-olds suggested some changes in volume of limbic structures (nucleus accumbens, amygdala, prefrontal cortex, rostral anterior cingulate cortex and hippocampus) associated with loneliness (Kiesow et al., 2020). In a Framingham cohort with 2,500 MRI scans, lower total brain volume and more white matter hyperintensities were associated with loneliness (Salinas et al., 2022). There was no significant association between loneliness and hippocampal volume. A small exploratory study of people with cognitive complaints revealed no relationship between loneliness and brain regions typically involved with AD (e.g., hippocampus and medial temporal lobe) (Zhang et al., 2022).

fMRI studies of loneliness in older adults are more limited (Lam et al., 2021). In one large study, 40,000 UK biobank participants with MRI scans showed a relationship between loneliness to midline subregions in the default network patterns and covariation between the hippocampus and dorsal network (Zajner et al., 2021). There is some suggestion that the functional connectivity changes associated with depression in older adults over frontal and temporal regions are different than the functional connectivity changes associated with loneliness in bilateral lingual gyri (Lan et al., 2015).

## 4 Mediators and moderators

As we have described, loneliness is associated with and is predictive of cognitive decline and dementia. Despite these findings, mechanisms of this relationship are unclear. The importance of identifying factors that may modify or underlie the relationship between loneliness and health outcomes has long been understood (MacKinnon and Luecken, 2008; National Academies of Sciences, Engineering, and Medicine, 2020). This body of work continues to expand, and many studies document the positive impact of protective factors such as social relationships and support (National Academies of Sciences, Engineering, and Medicine, 2020). However, despite the importance of this topic and recent increase in attention, mechanisms of the relationship between loneliness and negative health outcomes are still poorly understood (Boss et al., 2015; Yu and Siang Ng, 2023). Further, the studies that have explored these associations are primarily descriptive, and longitudinal mediation analyses are rare (Holwerda et al., 2014; Kim et al., 2020). Even more rare is consideration of the potential role of physiological, psychological, or social variables that may impact or account for this relationship (Holwerda et al., 2014).

A handful of studies have explored the potential buffering role of a healthy social network on the development of dementia and cognitive decline [e.g., Bennett et al. (2006), Karp et al. (2006), and Kuiper et al. (2015)]. Some such studies suggest that an active social life may protect against dementia (Fratiglioni et al., 2004).

Some preliminary work in this area has found that negative health conditions or behaviors linked to loneliness, such as depression (Van As et al., 2022), lack of exercise (Pels and Kleinert, 2016), and poor sleep (Griffin et al., 2020b) also increase the risk for negative cognitive changes (Liew, 2019; Ma et al., 2020; Whitty et al., 2020). Complicating the issue of mechanisms is the fact that, although a consistently significant and positive relationship between loneliness and general cognitive ability that has already been described, there are some large studies have partially contradicted these findings, at least in terms of time course [e.g., Okely and Deary (2018)]. Previous research has suggested that cultivating a better understanding of mechanisms at play may help to clarify these contradictory findings (Kim et al., 2020). Similarly, larger longitudinal epidemiological studies may shed some light on the relationship between loneliness and cognitive decline, but these studies may also be coincidental and not causative. For this reason, review of the intervention literature is needed to elucidate these relationships. Interventions for loneliness and their impact on cognitive functioning are discussed in below in section 5.

### 4.1 Depression

Various aspects of psychiatric health may be important in the relationship between loneliness and cognitive decline. An overview of 40 systematic reviews of loneliness and social isolation found they were associated with worse mental health outcomes (Leigh-Hunt et al., 2017), and a host of reports indicate that loneliness is associated with particular mental health symptomatology such as depression and anxiety (Hawkley and Cacioppo, 2010). The issue of depression is particularly important, as it is a known risk factor for cognitive decline and dementia (Almeida et al., 2017; Rubin, 2018; Elser et al., 2023), although the neuropathology of depression does not imply AD pathology (Wilson et al., 2014; Nunes et al., 2022). Similarly, loneliness is also a specific risk factor for depression (Heikkinen and Kauppinen, 2004; Cacioppo et al., 2006b). A recent systematic review and meta-analysis found that loneliness significantly predicted suicidal ideation and behavior, and that depression significantly mediated this relationship (McClelland et al., 2020). While most individual studies observing the cognitive decline associated with loneliness have adequately adjusted for depression, one study confirmed the relationship between loneliness and cognitive decline but the relationship was less clear after controlling for depression (Donovan et al., 2017).

Importantly, although social isolation and loneliness may coexist with depression, they are distinct (Taylor et al., 2018). It has been documented by previous studies that although loneliness is closely associated with depression (Cacioppo et al., 2010) and marital status (Theeke, 2009), most lonely older adults are married, cohabitating, and do not meet clinical criteria for depression (Perissinotto et al., 2012). Similarly, it has been reported that loneliness may precede depression chronologically. Loneliness is associated with higher increases in depression over time, rather than depression being associated with loneliness (Hawkley and Cacioppo, 2010). Although the findings generally support the distinction between depression and loneliness, other results are contradictory, particularly regarding the predictive relationships between loneliness, depression, and cognitive ability (Boss et al., 2015; Donovan et al., 2017). Most studies have found that loneliness predicts cognition beyond depression [e.g., Perissinotto et al. (2012), Holwerda et al. (2014), Freak-Poli et al. (2022), and Sutin et al. (2023)], while fewer others have found that the significant relationship between loneliness and cognition disappears when adjusting for depression (Gow et al., 2013; Donovan et al., 2017). Because of the complex relationships between depression, loneliness, and cognitive function, since depression may be a component of the mechanism of action in this relationship, it is important to demonstrate that the effect of loneliness on cognitive function is at least partly independent of depression (Wilson et al., 2021). In addition to psychiatric factors such as depression, other more static elements such as certain aspects of personality may also be mechanisms in the relationship between loneliness and cognitive decline.

### 4.2 Personality factors

One such personality trait that may play a role in the relationship between loneliness and cognition is neuroticism, which is described as persistent negative affectivity including anxiety, fear, and emotional instability (Thompson, 2008). Studies have shown that neuroticism is related to the development of MCI, dementia, and decline in episodic memory (Foong et al., 2018), and that higher neuroticism may be a risk factor for the development of dementia among older adults (Low et al., 2013; Kassem et al., 2018). Importantly, this association appears to be independent of AD pathology (Wilson et al., 2003, 2007a; Chapman et al., 2020; Franks et al., 2021).

Neuroticism has also been shown to be a primary personality factor associated with loneliness (Abdellaoui et al., 2019). Neuroticism may be somewhat unique among personality factors in this regard, as studies have shown that loneliness is negatively associated with extraversion and agreeableness (Mund and Neyer, 2019). Other research has also shown that neuroticism is associated with cognitive decline, and one study demonstrated that neuroticism mediated the relationship between loneliness and cognitive function in older adults (Foong et al., 2018). In an attempt to explain the mechanistic role of neuroticism, previous research has pointed out that lonely people are more likely to experience aspects of neuroticism, including anxiety, anger, and negative mood (Cacioppo et al., 2006a), which are associated with cognitive decline (Lindert et al., 2021). Foong et al. (2018) also suggested that their finding that neuroticism mediates the relationship between loneliness and cognitive decline may be due to older adults generally having weaker support systems and less outlets for internal stress.

Neuroticism is closely related to dysregulated physiological systems (Schneider, 2004; de Rooij et al., 2010; Evans et al., 2016; Brodersen and Lorenz, 2020; Wrzus et al., 2021) and physical health (Charles et al., 2008; Lahey, 2009), which have also been implicated as mechanisms of action in the relationship between loneliness and cognitive decline. In a study mentioned in the neuroimaging section above, the association between loneliness and gray matter volume changes in the left dorsolateral prefrontal cortex, a brain system closely related to physiological stress reactivity, was partially mediated by neuroticism (Kong et al., 2015).

In the Diagnostic and Statistical Manual of Mental Disorders [DSM-V; American Psychiatric Association (2013)], loneliness is not a diagnostic code but is considered a clinical symptom. Relatedly, loneliness is not considered a diagnosis in the International Statistical Classification of Diseases although that classification does include “problems related to living alone.”

### 4.3 Psychophysiological mechanisms

Multiple metrics of physical health have been suggested as potential mediators between loneliness and cognitive ability. These include objective measures such as individuals’ level of functioning, which is negatively associated with loneliness (Jakobsson and Hallberg, 2005) and positively associated with cognitive ability (Royall et al., 2012; Gavett et al., 2015). Global self-report measures of physical and psychological health have also been found to correlate with loneliness and cognitive ability (Bond et al., 2006; Luo et al., 2012). (Kim et al., 2020) posited that self-reported health may constitute a viable mechanism due to chronology: specifically, loneliness often precedes decline in self-rated health, and self-rated health decline often precedes cognitive decline (Tomaszewski Farias et al., 2018). Biomarkers of physiological systems have also been suggested to mediate the relationship between loneliness and cognition.

Although a recent report from the National Academy of Sciences concerning social isolation and loneliness identified physiological mechanisms that may be at play, they are largely theoretical in the current literature, and most studies analyzing the role of these mechanisms are based on cross-sectional data (Yu and Siang Ng, 2023). A prevailing theory seeking to explain the role of biomarkers in the relationship between loneliness and health outcomes is the Evolutionary Theory of Loneliness, which posits that the association between socialization and health is prescribed in human biology (Eisenberger and Cole, 2012; Cacioppo and Cacioppo, 2018). Other authors have expanded on this theory by suggesting that primitive humans who were lonely or isolated were more vulnerable than those who were more socially connected (Leschak and Eisenberger, 2019). If these individuals experienced activation of their fight-or-flight system more regularly than their counterparts, it is likely that they experienced associated physiological dysregulations (Cacioppo et al., 2003; Eisenberger and Cole, 2012).

Implicated physiological parameters include increased inflammation, renal injury, and poorer metabolic health (Cacioppo et al., 2003; Eisenberger and Cole, 2012). Research has also found that increased loneliness is associated with poorer functioning of the hypothalamic–pituitary–adrenal axis (Pressman et al., 2005; Cole, 2008) and cardiovascular systems (Cacioppo et al., 2002b; Momtaz et al., 2012). These physiological systems also represent significant risk factors for cognitive dysfunction (Yaffe et al., 2014; Walker et al., 2019; Nair et al., 2020), thereby increasing the risk for lonely older adults to experience cognitive impairment. Further research has supported this potential mechanistic pathway by also demonstrating that impairment of these physiological systems is also specifically associated with worse cognitive abilities (Barnes et al., 2003; Teunissen et al., 2003; Craft et al., 2012; Borsini et al., 2015; Nation et al., 2018). Finally, recent studies suggest that physiological dysregulation may theoretically serve as a primary mechanism in the relationship between loneliness and cognitive functioning (Shankar et al., 2013; Cacioppo et al., 2014a,b; Boss et al., 2015) (Kim et al., 2020) presents the example that loneliness is predictive of increased inflammatory responses (Cole, 2008) which in turn are related to increased stress and worsened cognition (Boss et al., 2015). Despite these promising findings, the field is generally conflicted regarding the mechanistic role of physiological markers in the relationship between loneliness and cognition. For example, a recent study examined numerous biomarkers, including glycosylated hemoglobin, low density lipoprotein and high density lipoprotein, C-reactive protein, and Cystatin C, none of which mediated the association between loneliness and cognitive impairment (Yu and Siang Ng, 2023). Despite uncertainty in the field, some research has delved deeper into the role of physiology in the relationship between loneliness and cognitive decline. A specific aspect of physiological dysregulation that has received attention and may mediate the relationship between loneliness and cognitive decline is impaired stress reactivity.

### 4.4 Stress reactivity

Loneliness has been related to aspects of stress reactivity such as increased sensitivity to and surveillance of social threats with biased responding toward negative social information (Cacioppo et al., 2009). In addition to psychosocial presentations, there is evidence that loneliness is associated with increased physiological stress and stress reactivity (Hawkley and Cacioppo, 2010; Boss et al., 2015; Cacioppo et al., 2015a). A 2017 systematic review investigating this relationship (Brown et al., 2018) reported that most included studies described a positive link between loneliness and acute physiological stress reactivity indices such as blood pressure (Steptoe et al., 2004; Nausheen et al., 2007; Ong et al., 2012), total peripheral resistance and pre-ejection period (Cacioppo et al., 2002b), and a variety of neuroendocrine markers such as IL-6 and IL-1β (Steptoe et al., 2004; Hackett et al., 2012; Jaremka et al., 2013). However, some studies reported nonsignificant (Steptoe et al., 2004; O'Donovan and Hughes, 2007) or inverse associations with cardiac output and heart rate (Cacioppo et al., 2002b), heart rate variability (Norman et al., 2011; Roddick and Chen, 2021), and likelihood of excessive neuroendocrine activity such as cortisol response (Hackett et al., 2012). Other studies have reported similar findings regarding cortisol, including that excessive stress reactivity is associated with alterations in cortisol and hypothalamic–pituitary–adrenal axis, and that increased cortisol is in turn associated with loneliness in young (Pressman et al., 2005) and older adults (Schutter et al., 2017). Notably, a recent study suggested that bedtime cortisol mediated the otherwise null relationship between loneliness and several aspects of cognitive functioning (Montoliu et al., 2019). The relationships between loneliness, cortisol, and other physiological systems have also been explored, largely in a theoretical capacity. Hawkley and Cacioppo et al. (2010) discuss the connection between cortisol and inflammation, and suggest that theoretically cortisol should produce an anti-inflammatory effect through activation of the glucocorticoid receptor. However, it is well-established that loneliness and social isolation are linked to increased risk for dysregulated inflammation. These authors suggest that this may be because these individuals experience glucocorticoid insensitivity, thereby allowing inflammation to escalate without a mediating process. Other research has confirmed the relationship between loneliness and inflammatory genes.

Canli et al. (2017) found that loneliness was associated with about 16,000 differentially expressed genes linked to neurological and psychological disease, and a variety of physical disorders. These authors specifically examined the nucleus accumbens, and their findings suggest potential mechanisms for future studies of gene networks in this area of the brain in the relationship between loneliness and related disorders. Neurobiology related to loneliness received attention via a recent systematic review, although most included individual studies were among young adults (Lam et al., 2021). Some of the neuroimaging data in older adults has been discussed section 3.3.3.

Research has also demonstrated relationships between physiological indices of stress reactivity and cognitive impairment. A recent systematic review found that a host of indices of blunted physiological stress reactivity was associated with lower cognitive ability (Turner et al., 2020). Specifically, individual studies demonstrated that blunted cardiovascular reactivity (e.g., lower heart rate reactivity) was related to lower cognitive ability (Ginty et al., 2011; Yano et al., 2016). Other blunted physiological stress reactions have been associated with lower cognitive performance, including cortisol (Ginty et al., 2012). Loneliness may also play a role as a moderator of the relationship between hormones and peripheral nervous system activity. A 2011 study found that loneliness was significantly related to the relationship between oxytocin and sympathetic cardiac control.

### 4.5 Other potential mediators or moderators

It is well-established in the literature that social isolation and loneliness negatively impact health-related behaviors such as smoking or physical activity which in turn relate to cognitive function. In addition to cognitive function, the interaction between loneliness and health-related behaviors may have additional downstream effects on more general health outcomes. For example, one study found that negative health behaviors such as low physical activity, regular smoking, and poor sleep mediated the relationship between loneliness and poor health outcomes (Christiansen et al., 2016). Poor sleep in particular has received attention as a possible link between loneliness and cognitive function. It has been pointed out in previous research that since poor sleep is related to both worse cognitive function and loneliness, it is one potential mediator of the effect of loneliness on cognition (Shankar, 2020).

Studies have also considered systemic physiological markers in the relationship between loneliness and health outcomes. A recent study evaluated the mediating role of an aggregate allostatic load index to indicate multisystemic physiological risk. Allostatic load is defined as the cumulative burden of chronic stress (Guidi et al., 2021). This variable included cardiovascular functioning as indicated by systolic and diastolic blood pressure, and pulse rate. Also included was C-reactive protein as a measure of inflammatory functioning, glycosylated hemoglobin as a measure of metabolic functioning, a marker for Epstein–Barr Virus antibodies as an indicator of immune functioning, dehydroepiandrosterone-sulphate as a measure of neuroendocrine functioning, and body mass index as a measure of anthropometric health. Despite theoretical support, the authors found that this aggregate variable did not mediate the relationship between loneliness and cognitive functioning (Kim et al., 2020).

Although the relationships between loneliness, cognitive functioning, and depression have been explored, other psychiatric conditions have also been proposed as potential mechanisms of the relationship between loneliness and cognitive functioning. Prior research in this area has found that in addition to increasing depressive symptoms, loneliness also increases anxiety (Cacioppo et al., 2006a). Anxiety has also been suggested to play a role in a self-sustaining cycle in which negative social experiences cause lonely people to further distance themselves from others (Newall et al., 2009). This cycle is accompanied by compounding feelings of anxiety, which may in this way be serving some mechanistic role between loneliness and negative outcomes. Despite the frequent co-occurrence depression and anxiety, a study comparing the mechanistic roles of these conditions in a large sample of older Irish adults found that depression, but not anxiety mediated the relationship between loneliness and cognitive function (McHugh Power et al., 2020).

A 2021 study sought to investigate potentially modifiable psychological factors that might influence the relationship between loneliness and risk for dementia (Yang et al., 2021). These authors conducted a moderated mediation analysis, through which they found that a sense of control significantly mediated the relationship between loneliness and risk of dementia. However, per the authors’ analyses, this was only true for individuals with poorer working memory capacity. This suggests individual sense of agency and improvement in specific cognitive domains may be highly relevant treatment targets for healthy aging in older adults. Another recent study among older Chinese adults found that the relationship between loneliness and cognitive function was significantly moderated by internet use, such that the negative impact of loneliness on cognitive function was more detrimental among those who used the internet less (Li et al., 2022b). These findings suggest that certain social outlets may also serve as treatment targets.

## 5 Treatment targets

Interventions to address loneliness in older adults vary and have had limited success because the theoretical underpinnings of loneliness are poorly understood (Findlay, 2003; Dickens et al., 2011; Masi et al., 2011;O'Rourke et al., 2018; Akhter-Khan and Au, 2020). Despite the complexity and heterogeneity of loneliness, key targets for intervention are often singularly focused on engagement in one’s social network (O'Rourke et al., 2018; Akhter-Khan and Au, 2020). Indeed, interpersonal contact is not sufficient to address chronic loneliness in the general population (Cacioppo et al., 2015c). Given the lack of consistent relationship between loneliness and social isolation, this discussion tries to focus on interventions that decrease negative emotional responses or stress to social interactions. Rather than taking a one-size-fits-all approach, recent scholarship points to the need for theory-driven interventions that consider key treatment targets and the context of the individual (O'Rourke et al., 2018; Akhter-Khan and Au, 2020; Van Orden et al., 2021). For example, the subjective experience of loneliness may be addressed through targeting maladaptive cognition (i.e., hypervigilance to negative social evaluation) with cognitive behavioral therapy (Hawkley and Cacioppo, 2010; Masi et al., 2011), and the objective experience of loneliness may be addressed through social skills training and supported social engagement (Akhter-Khan and Au, 2020). Many interventions in older adults target social isolation without a clear focus on loneliness *per se* and are not discussed. The issue that many interventions not targeting loneliness coincidentally have a significant social component are also not discussed, e.g., the social support environment may represent an important feature of exercise programs for improving older adults’ perceived loneliness (Ehlers et al., 2017). Digital technology interventions and social media platforms may be useful in an older population but are also not discussed in detail due to additional limitations of generalizability and the lack of many high-quality studies targeting loneliness (Welch et al., 2023). Here, we discuss interventions for loneliness in older adults with key treatment targets that have the potential to optimize cognitive function in older adults.

### 5.1 Cognitive control as a treatment target

Loneliness is characterized by attentional bias to socio-affective stimuli, particularly negative social evaluation, resulting in diminished capacity for self-regulation (Hawkley and Cacioppo, 2010; Wong et al., 2022). Self-regulation, which is the ability to control and generate cognitive, emotion, and physiological responses that support goal-directed behavior, is essential to health and adaptability. Results from a recent meta-analysis provide proof-of-concept that loneliness up-regulates cognitive control networks to process socio-affective information (Wong et al., 2022). Integrated models of stress adaptation point to shared neural underpinnings for regulation of cognition, emotion, and physiological stress (Hawkley and Cacioppo, 2010), suggesting that targeting cognitive control may impact emotion dysregulation in loneliness.

Mindfulness-based interventions offer an approach to target mechanisms driving loneliness, specifically cognitive control and emotion regulation. Mindfulness training improves cognitive domains (Tang et al., 2007; Goldin and Gross, 2010; Fjorback et al., 2011; Hölzel et al., 2011; Desrosiers et al., 2013; Prakash et al., 2015; Tang et al., 2017) including attention and cognitive control, which are notably deficient among lonely individuals (Wong et al., 2022). Training attention has been shown to be an important mechanism for self-regulation of cognition and emotion, both of which allow for context appropriate responses (Goldin and Gross, 2010). Specifically, mindfulness training disrupts rumination, which has the potential to reduce hypervigilance for negative social evaluation (Li et al., 2022a). Mindfulness-based interventions train participants to attend to the present moment without judgment, which may teach participants to engage in a novel, regulated response, like cognitive reappraisal, rather than a habitual or automatic response, like negative social expectations (Hawkley and Cacioppo, 2010). Initial evidence from a mindfulness-based stress reduction intervention showed reductions in loneliness among older adults, including significant down-regulation of the expression of NF-κB-associated gene expression profile, which are upregulated in lonely older adults (Creswell et al., 2012). Mindfulness has a beneficial impact on both acute physiological stress reactivity and cognitive ability (Singh et al., 2012).

### 5.2 Maladaptive cognition as a treatment target

Maladaptive social cognition is associated with loneliness, including attentional bias toward negative aspects of social experiences. Attentional bias toward social threat may confirm one’s beliefs about social interactions and perceived loneliness, which may reinforce behaviors like social withdrawal (Cacioppo et al., 2015c). Addressing maladaptive social cognition to reduce emotional loneliness is an important treatment target (Masi et al., 2011; Akhter-Khan and Au, 2020; Van Orden et al., 2021). Cognitive behavioral therapy trains individuals to identify, challenge, and reframe automatic thoughts associated with self, others, and social experiences, that may be maladaptive, thereby exacerbating feelings of loneliness and influencing behaviors. Further, cognitive behavioral therapy teaches individuals to look for evidence of social connectedness to help decrease biased cognitions. In a meta-analysis of interventions to reduce loneliness, interventions which addressed maladaptive social cognition was larger (moderator analysis) than for interventions to improve social skills, enhance socials support, or increase opportunities for social interaction (Masi et al., 2011).

### 5.3 Heart rate variability as a treatment target

Heart rate variability (HRV) as a measure of cardiac vagal tone (Laborde et al., 2017) is another potential treatment target for loneliness. An indicator of autonomic nervous system function, HRV measures the fluctuation of the length of heartbeat intervals and is used to index central control of the heart via the vagus nerve. Indeed, cognitive control, emotion regulation, and HRV are associated with shared brain regions, including the ventromedial prefrontal cortex and amygdala. The neurovisceral integration model suggests that HRV is a marker of self-regulatory capacity, serving as a peripheral index of the integrity of these neural structures to exhibit inhibitory control and generate cognitive, emotion, and physiological responses that support goal-directed behavior, which is essential to health and adaptability (Thayer et al., 2009). High-frequency HRV is an indicator of strong vagal parasympathetic regulation of the heart, which correlates to increased attentional control, emotion regulation, and social engagement, all of which are markedly diminished in loneliness (Porges and Furman, 2011). Reduced HRV is an indicator of low capacity for stress adaption. Although loneliness is characterized by diminished capacity for stress adaptation and self-regulation, empirical support for the link between HRV and loneliness is emerging, with a recent study supporting evidence of chronic loneliness predicting lower resting HRV (Roddick and Chen, 2021). HRV biofeedback is a non-invasive behavioral intervention that trains individuals to modify respiratory rate in a way that maximizes respiratory sinus arrhythmia, which is the variation in heart rate during the breathing cycle and a peripheral marker of cardiac-linked parasympathetic regulation. HRV biofeedback interventions are promising for targeting both cognitive function and loneliness among older adults. A preliminary three-week HRV biofeedback intervention was conducted among older adults and results indicated improvements in attentional skills (Jester et al., 2019). Another study examined HRV biofeedback and loneliness in older adults and provided evidence that HRV biofeedback improved loneliness among institutionalized older adults (de Souza et al., 2022).

## 6 Discussion

Loneliness is a common condition in older adults, and it has a significant relationship with many poor health outcomes, including mortality. Importantly, loneliness is also a significant risk factor for the development of cognitive decline and dementia. As discussed, the risk of loneliness contributing to development of dementia in longitudinal studies (HR 1.61) is on a level not much lower than the risk of having a single APOE4 gene (HR 1.75). Worse cognition in longitudinal studies has been fairly consistently related to loneliness, even after adjusting for potential confounds such as depression. Of note, one large epidemiological study even found that mid-life persistent loneliness increased the risk of cognitive decline later in life (Akhter-Khan et al., 2021), suggesting that persistent loneliness may be a trait issue. Global cognition encompassing clinical diagnosis of MCI or dementia is the usual outcome measure used in relevant studies and it has demonstrated a consistent relationship with loneliness. Fewer studies use a broad cognitive assessment battery. The cognitive domain literature is not completely consistent, but the data suggest that episodic memory is not the main domain impacted by loneliness. To further weigh in on the hypothesis that cognitive change is not related to AD brain changes, several longitudinal studies looking at post-mortem neuropathology found no relationship between loneliness and AD pathology (Wilson et al., 2007b; Jackson et al., 2023). This lack of AD pathology is similar to what is observed with chronic stress, i.e., neuroticism, where cognitive change or dementia is greater than in controls but there is no clear relationship to AD pathology (Wilson et al., 2003, 2007a; Chapman et al., 2020; Franks et al., 2021). This lack of AD pathology even extends to neuroimaging where medial temporal lobe changes have not been consistently related to loneliness, even as other areas have demonstrated changes (Salinas et al., 2022; Zhang et al., 2022). While not completely consistent, there is evidence that the risk of loneliness contributing to cognitive decline or dementia may be greater in those with fewer conventional risks of AD dementia, such as lower age or being APOE4 negative.

There are a number of potential mediators of the relationship between loneliness and cognitive decline and dementia. One particularly complex potential mechanism is depression, which is associated with loneliness and with development of dementia. These relationships are further complicated by the inclusion of questions about loneliness on depression screening scales such as the CES-D. However, many longitudinal studies have adequately controlled for depression and still observed the relationship between loneliness and cognitive deficits, so depression is not the only mediator. Excessive stress reactivity has been noted in loneliness and is a known risk factor for development of cognitive decline and dementia. One clinical marker for excessive stress reactivity, neuroticism, has also been linked to loneliness and the development of dementia (see section 4.2 above). Despite promising evidence for factors that may underlie or influence the relationship between loneliness and cognitive decline, more research is needed to clearly identify constructs at play so that they may be integrated into effective treatments.

The interacting bidirectional factors of dementia, depression, and loneliness are particularly hard to tease apart. The relationship between loneliness and cognitive change is a complex network involving many interacting collinear factors with temporal relationships that are not clear or consistent (e.g., loneliness causing depression, depression causing loneliness, both contributing to cognitive decline, and cognitive decline contributing to both). Other potential confounds that may inter-relate with loneliness and cognition include physical activity, SES, sex, marital status, and general health. These relationships suggest that future analyses would benefit from more complex causal analyses based on structural equation modeling incorporating time (Hoyle, 2023) or a systems science modeling approach (Oken et al., 2015; Mobus, 2022; Hoyle, 2023).

Some interventions that may decrease cognitive decline, such as exercise may exert its effect at least in part through social interactions, resulting in decreased loneliness that is often a part of exercise group interventions (McAuley et al., 2000). However, our understanding of mechanisms of exercise improving cognitive health is made even harder to describe, since loneliness may contribute to decreased exercise and decreased physical activity (Hawkley et al., 2009).

There is a concern that some of the observed relationships between loneliness and cognitive decline may be related to decreases in social interactions related to onset of dementia (based on longitudinal studies). This confound has the potential to result in reverse causality in cross-sectional studies, already noted in section 3.1 and for further details see discussion in another paper (Zhou et al., 2018). Some longitudinal studies appropriately even exclude people with dementia onset shortly after study enrollment while still apparently cognitively healthy, even up to within 5 years of starting a 20-year longitudinal study (see Table 1).

While every effort was made to search for articles systematically and thoroughly, this is not a systematic review. We retrieved all identified articles that were available from a wide variety of journals and included both quantitative and qualitative work. Importantly, we analyzed all references in recently published relevant systematic reviews. There is, however, the usual bias toward work that is published in English.

Another limitation of the current review is that all papers used a simple assessment of loneliness. In the papers reviewed, there was no attempt to divide loneliness into subtypes discussed in the introduction, i.e., emotional or intimate loneliness (absence of meaningful relationships), social loneliness (perceived deficit in quality of social connections), and existential loneliness (feeling of fundamental separateness from others and the wider world). These loneliness subtypes may have differential effects on cognitive decline, and this may be a useful point to explore in future research.

The longitudinal studies evaluating loneliness all predate the newer consensus criteria for AD currently being finalized that incorporate biomarker information (Alzheimer’s Association International Conference, 2023), so the common usage of the term “Alzheimer’s Disease” in the literature is most often outdated. The neuropathological and other evidence that the dementia related to loneliness is not caused by AD pathology has resulted in our usage of the term “dementia.”

Since all the studies investigated slightly different variables in different groups of patients, it was not possible to undertake a combined quantitative analysis. Nevertheless, this review provides an up-to-date discussion of what influences the relationship between loneliness and cognition in older adults and identifies areas where further work would be valuable.

The review of potential treatment targets for loneliness among older adults is not an exhaustive search of all possible evidence. Instead, we identified key treatment targets for loneliness (i.e., cognitive control, maladaptive cognition, and heart rate variability) that have the potential to enhance interventions to optimize the decline in older adults’ cognitive function related to loneliness. The treatments selected (i.e., mindfulness-based interventions, cognitive behavioral therapy, and heart rate variability biofeedback) are interventions that target mechanisms of action to optimize cognitive function and have evidence of improvement in loneliness among older adults. We have not discussed interventions to simply decrease social isolation since, as already discussed, social isolation is relatively independent of loneliness and the discussion focused on the feelings that contribute to the sense of loneliness.

In summary, loneliness significantly contributes to cognitive decline, as well as other health problems. Loneliness is a factor that needs to be used as an adjustment when evaluating cognition or dementia in epidemiological or clinical studies in older adults. For example, while the data on exercise associated with decreased incidence of dementia is very strong, the amount of human social interactions in cross-sectional, longitudinal, or intervention studies of exercise in aging is often not well controlled, and lonely people are less likely to exercise. In general, development of targeted intervention strategies to decrease feelings of loneliness in older adults will be useful to decrease the risk of cognitive decline. Multimodal interventions to decrease cognitive decline in older adults should include interventions to reduce loneliness and not just social isolation. There may also be tertiary factors associated with both loneliness and cognitive decline that may serve as novel treatment targets. It will continue to be important to explore the potential mediators of the effect of loneliness on cognition in older adults, even knowing that this will be difficult because of the overlapping established correlates of both loneliness and cognitive decline.

## Author contributions

BO: Writing – original draft, Writing – review & editing, Conceptualization, Supervision, Visualization. JK: Writing – original draft, Writing – review & editing. DK: Writing – original draft, Writing – review & editing, Visualization. AG: Writing – original draft, Writing – review & editing.
